# Yak Milk: Nutritional Value, Functional Activity, and Current Applications

**DOI:** 10.3390/foods12112090

**Published:** 2023-05-23

**Authors:** Diandian Wang, Yaxi Zhou, Xianping Zheng, Jinhong Guo, Hao Duan, Shiqi Zhou, Wenjie Yan

**Affiliations:** 1 College of Biochemical Engineering, Beijing Union University, No. 18, Xili District 3, Fatou, Beijing 100023, China; spwdd2018@163.com (D.W.); 15239407080@163.com (Y.Z.); gjh121133@163.com (J.G.); dhuanao@163.com (H.D.); shiqizhougood@163.com (S.Z.); 2 Beijing Key Laboratory of Bioactive Substances and Functional Food, College of Biochemical Engineering, Beijing Union University, 197 North Tucheng West Road, Beijing 100023, China; 3 Ganzi Prefecture Seda County Zang Yuan Zhao Mei Dairy Products Co., Ltd., No. 51, West Section of Jinma Avenue, Seda County, Ganzi Tibetan Autonomous Prefecture, Ganzi 626700, China; 18937015122@163.com

**Keywords:** yak milk, ingredients, nutrition, function, application

## Abstract

The yak is a special species that inhabits the Qinghai-Tibet Plateau and its surrounding areas. Its unique habitat gives yak milk certain distinct characteristics compared to regular cow milk. Yak milk not only has a high nutritional value but also holds potential benefits for human health. In recent years, there has been increasing research attention on yak milk. Studies have found that the bioactive components in yak milk have various functional properties, including antioxidant, anticancer, antibacterial, blood pressure-lowering, anti-fatigue, and constipation-relieving effects. However, more evidence is needed to confirm these functions in the human body. Therefore, by reviewing the current research status on the nutrition and functionality of yak milk, we aim to reveal its enormous potential as a source of nutritional and functional substances. This article primarily analyzed the nutritional composition of yak milk and the functional effects of its bioactive components, categorically elucidated the mechanisms behind its functional activities, and provided a brief introduction to related yak milk products. Our objective is to deepen people’s understanding of yak milk and provide some references for its further development and utilization.

## 1. Introduction

**Figure 1:**
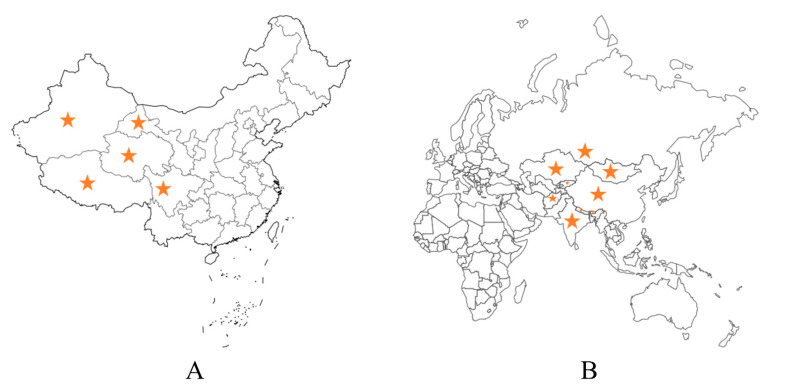
Distribution of yaks in China (**A**) and around the world (**B**). The stars in the figure represent the main habitats of yaks.

The yak (*Bos grunniens*) is a unique member of the bovine family, as it is able to survive and reproduce in harsh environments. The majority of wild yaks are found in the Himalayan region at altitudes exceeding 3000 m [1]. This is because yaks are well-adapted to extreme conditions, including cold temperatures, low pressure, hypoxia, strong UV radiation, large temperature variations, and food scarcity [2,3]. In China, yaks mainly inhabit several provinces on the Qinghai-Tibet Plateau, such as Xinjiang, Gansu, Qinghai, Sichuan, and Tibet, which are all considered the yak’s natural habitat. There are approximately 15 million yaks living on the Qinghai-Tibet Plateau, which accounts for more than 90% of the worldwide yak population [4]. Yaks are also found in neighboring countries around China, such as Mongolia, India, Bhutan, Nepal, Afghanistan, Pakistan, Kyrgyzstan, and southern Russia, which are all located in high-altitude areas of Central Asia and closely related to the yak’s preferred habitats [5]. Figure 1 shows the distribution of yaks in China and around the world. 

Yaks play an important role in human survival in high-altitude areas, as they provide essential items such as yak milk, meat, hide, and fuel (dried yak dung) for the local people [6]. Moreover, yaks are frequently used as a source of power for transportation in high-altitude areas. Yak milk can be used directly or processed into various traditional foods such as butter, cheese, yogurt, and Tibetan tea by local herdsmen. Compared to other types of milk, yak milk appears to contain a richer and more diverse range of nutrients. During its primary lactation period, yak milk has high levels of fat (5.5–7.5%), protein (4.0–5.9%), and lactose (4.0–5.9%). It is considered a naturally concentrated milk [7]. Therefore, yak milk is one of the main economic sources of milk in China, second only to cow milk and buffalo milk [8]. The unique amino acids, fatty acids, high levels of vitamins, specific enzymes, and beneficial microorganisms found in yak milk may have a positive impact on the health of nomadic populations in high-altitude regions [9]. The latest review paper suggests that yak milk and its derivatives possess various bioactive functionalities, including antioxidant, anticancer, antimicrobial, blood pressure-lowering, anti-fatigue, and constipation treatment properties. These findings open up possibilities for the high-value utilization of yak milk [10].

In this paper, we reviewed the nutritional composition of yak milk, categorized the functional effects and exerting mechanisms of yak milk, and briefly summarized the related products of yak milk to provide some reference for the in-depth development and utilization of yak milk.

## 2. Nutritional Components of Yak Milk

### 2.1. Substance Composition of Yak Milk

The composition of yak’s milk varies during different periods, with high amounts of fat, protein, and lactose present during its main lactation period [11]. The composition of yak milk also differs based on the season and the number of pregnancies. Unsaturated fatty acids are higher in yak milk during the summer compared to the winter. In addition, multiparous yaks (2nd to 5th pregnancies) have higher levels of monounsaturated fatty acids and polyunsaturated fatty acids in their milk fat (31.61 and 4.20 g/100 g total fatty acids, respectively) compared to primiparous yaks [12]. Furthermore, hybrid yak breeds exhibit better milk production and higher fat content than regular yak breeds [13]. Additionally, the colostrum of yaks contains significantly higher levels of solids, protein, and fat compared to non-colostrum milk. The solid content in yak colostrum is twice as much as that in non-colostrum milk; the protein content is three times higher; and the fat content is two to three times higher [14,15]. The ash content in yak milk is similar to regular cow milk, ranging from approximately 0.7 to 0.9, and it is not influenced by seasonal changes [7]. Table 1 shows the composition of yak’s milk in different breeds, which shows that although the composition of different yak’s milk varies, there is not much overall difference, with the milk solids content accounting for about 16–18%, milk fat content for about 5.6–7.5%, protein content for about 4.7–6.5%, lactose content for about 3.5–5.5%, and ash content for about 0.77–0.95% [16].

### 2.2. Yak Milk Protein

Protein is one of the main nutritional substances in yak milk, with a content of about 4.5% to 6.5%. The protein content in yak milk is affected by breed, feed, parity, lactation stage, and season. The protein content in yak milk directly affects the physicochemical properties and nutritional value of yak dairy products, such as density, viscosity, surface tension, and amino acid score.

#### 2.2.1. Distribution of Nitrogen in Yak Milk

The nitrogen composition of milk can be divided into three categories: casein nitrogen (CN), whey protein nitrogen (WPN), and non-protein nitrogen (NPN). The distribution of nitrogen in yak milk varies by season, with a higher total nitrogen (TN) content in the milk during warmer seasons [7]. Generally, as the environmental temperature increases, the total protein content in milk decreases, and this abnormal phenomenon may be related to the scarcity of food during the winter [18,19]. Table 2 shows the nitrogen distribution in different types of yak milk. From the table, it can be seen that there is a big difference in the NPN content among different breeds of yak milk, while the content of WPN and CN does not vary much. It is worth noting that the ratio of WPN to TN in yak milk is higher than in regular milk (around 17%). This may be one of the reasons why yak milk exhibits strong functional activity [19,20]. The NPN in yak milk mainly comes from free amino acids: urea, uric acid, peptides, creatine, creatinine, lactic acid, and ammonia [21].

#### 2.2.2. Protein Composition of Yak Milk

Similar to other milk sources, the proteins in yak milk are mainly divided into two categories. One is casein, including αS1-casein, αS2-casein, β-casein, and κ-casein. The other is whey protein, including α-lactalbumin, β-lactoglobulin, serum albumin, lactoferrin, and immunoglobulins. Table 3 shows the types and contents of proteins in different milks. As shown in Table 3, the content of the four types of casein in yak milk is similar to that in goat milk. Except for αS1-casein, the content of the other three types of casein in yak milk is higher than that in cow milk. The peptides released by casein in yak milk have multiple biological activities, suggesting that yak milk has stronger potential functional activity than ordinary cow milk [24,25]. Whey protein is a good source of essential amino acids in the diet. The whey protein content in yak colostrum is higher and richer in various proteins related to immune function [14]. The data in Table 3 shows that yak milk contains a higher content of total whey protein. It is worth noting that the content of β-lactoglobulin in yak milk is higher than that in other milks, accounting for about 65% of the total whey protein in yak milk.

#### 2.2.3. Amino Acid Composition of Proteins in Yak Milk

The amino acid content in yak milk is also influenced by the season, mainly due to the different food sources for yaks in different seasons [16]. Table 4 shows the amino acid composition of different types of milk. It is certain that the total amino acid content and most of the amino acid content in yak milk are higher than those in cow, sheep, and human milk because the protein content in yak milk is higher. As shown in Table 4, the EAA/NEAA ratio in yak milk is 73% and the EAA/TAA ratio is 42%, which fully meets the recommendation of FAO/WHO of 60% and above 40%.

### 2.3. Fat

The fat content in yak milk ranges from 5.6–7.5%. The fat and fatty acid composition of yak milk varies with the seasons. Studies have found that milk production is highest in yaks during autumn and summer, and the unsaturated fatty acid content in yak milk is higher in these seasons compared to winter [27]. This may be due to the abundance of fresh grass during the summer and autumn. Comparing different regions and breeds of yaks, it was found that the cholesterol content in yak milk ranged from 12.32–16.17 mg/100 g and was positively correlated with the fat content in yak milk [28]. Using the GC-MS method, a study analyzed the content of 69 fatty acids in different milks. Results showed that yak milk had high amounts of odd and branched-chain fatty acids and a lower ratio of n-6 to n-3 polyunsaturated fatty acids (PUFAs) [29]. Although the content of short-chain and medium-chain fatty acids in yak milk was lower than in cow’s milk, yak milk was rich in long-chain and unsaturated fatty acids, especially PUFAs [9,30]. Although it does not match up to human milk in terms of content, yak milk has a higher nutritional value compared to other types of milk. Therefore, yak milk is considered a potential functional food for human consumption. Table 5 shows the detailed fatty acid composition of yak milk.

### 2.4. Minerals

The content of minerals in yak milk is related to environmental factors. Research has found that the higher the altitude of the environment where yaks live, the higher the content of Mn and Fe in yak milk [33]. Additionally, there are significant differences in mineral content in yak milk from different breeds. Table 6 shows the mineral content of different sources of milk. It can be seen from the table that, except for P, the content of minerals in yak milk is much higher than that in bovine milk. The content of calcium and phosphorus in yak milk is much higher than that in human milk, approximately five times and three times, respectively. Yak milk contains more iron than regular cow’s milk, and the iron content is higher in Gannan yak milk than in Maiwa yak milk. This is beneficial for infants because the lower iron content in regular cow’s milk is the cause of iron deficiency in infants after drinking milk [34]. In addition, Maiwa yak milk has the highest content of Zn, which may serve as a good source of Zn for infant diets.

### 2.5. Vitamins

Table 7 shows the composition and content of different vitamins in milk. Clearly, yak milk contains higher amounts of Vitamin B_6_ and Vitamin D, which may be due to the fact that yaks generally live in high-altitude environments with long exposure to ultraviolet radiation. The vitamin content in yak milk varies greatly among different breeds and differs from that of regular cow’s milk. Seasonal variations in yak’s food sources lead to changes in the vitamin content of yak milk. Yak or dairy cows raised on artificial feed contain higher amounts of vitamins [16]. Recent studies have also found that yak milk contains 32.8 mg/L of Vitamin C, and this content increases with altitude [31,33].

### 2.6. Other Nutritional Components

In addition to the above components, yak milk also contains sphingolipids, phospholipids, and some oligosaccharides [36,37]. These substances are not only beneficial for the growth and development of yak calves but also have certain health benefits for human bodies. There are also a large number of beneficial probiotics for human health in yak milk, such as *Lactobacillus rhamnosus* CY12 [38], *Lactobacillus plantarum* strain As21 [39], and *Kluyveromyces marxianus* PCH397 [40]. These probiotics and their metabolites have tremendous potential for promoting human health.

## 3. Functional Properties of Yak Milk

**Figure 2:**
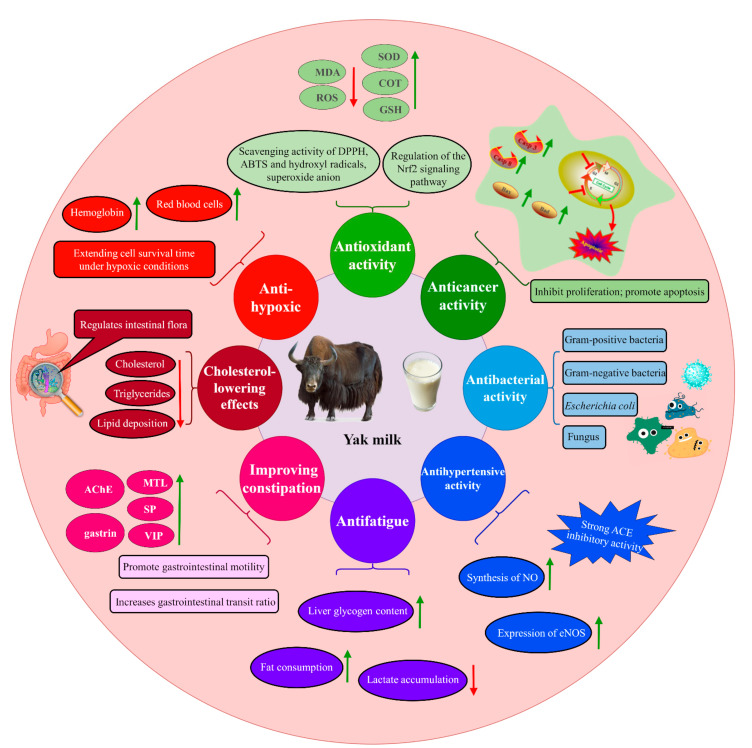
The potential mechanisms and impacts of yak milk components on improving human health conditions.

Yak milk is not only rich in nutrients but also contains various active components that endow it with multiple functional properties. Extensive in vitro and in vivo studies have found that yak milk and its bioactive components possess antioxidant, anticancer, antibacterial, blood pressure-lowering, anti-fatigue, and constipation-improving effects. This provides potential for the development of functional products using yak milk. However, there is currently a lack of sufficient clinical trials to confirm the therapeutic effects of these functionalities in the human body. In the following chapters, we have compiled and categorized the functional activities of yak milk, along with their corresponding mechanisms, and will elaborate on them. Figure 2 demonstrates the functional role of yak milk.

### 3.1. Antioxidant Activity

Antioxidants have attracted the attention of the scientific community due to their ability to prevent various degenerative diseases [41]. Natural antioxidants have been extensively studied for their non-toxicity and greater therapeutic effects on human health [42]. The antioxidant activity of yak milk is attributed to the biologically active peptides present in its protein and the lactic acid bacteria isolated from its naturally fermented milk. One study used alkaline protease and trypsin to hydrolyze yak casein to prepare yak casein hydrolysate (YCH), which was purified and tested in vitro for its ability to scavenge superoxide anions, hydroxyl radicals, and 2,2-diphenyl-1-picrylhydrazyl (DPPH) radicals, as well as its reducing power and chelating ability towards ferrous ions. The purified biological peptides were found to have higher antioxidant activity, indicating that highly active antioxidant peptides can be isolated and purified from yak casein, providing a pathway for researchers to obtain naturally sourced antioxidant peptides [43]. Furthermore, another study evaluated the antioxidant properties of yak milk whey protein hydrolysate peptide T10 in a damage model of hydrogen peroxide-induced human umbilical vein endothelial cells (HUVECs), which showed significant alleviation of cell damage, increased cell survival rates, and regulation of protein expression in the apoptotic genes bcl-2 and bax and the Nrf2 pathway to inhibit cell apoptosis and exert antioxidant activity [44]. In addition, a study on the antioxidant properties of lactic acid bacteria in fermented yak milk found that *L. delbrueckii* F17, isolated from fermented yak milk, significantly increased glutathione (GSH) and catalase (CAT) activity in the liver and serum of D-galactose-induced aging mice, as well as superoxide dismutase (SOD) activity in serum and the brain, while significantly reducing malondialdehyde content, indicating its strong antioxidant ability [45]. Additionally, *Lactobacillus plantarum* As21 isolated from fermented yak milk also exhibited antioxidant activity in the intestine of Caenorhabditis elegans and may be a potential probiotic strain for delaying aging [39]. In conclusion, biologically active substances and lactobacillus strains isolated and purified from yak milk can effectively regulate oxidative stress reactions and serve as a promising source of natural antioxidants for the future development and use of functional foods and drugs.

### 3.2. Anticancer Activity

Currently, there are many drugs available for cancer treatment, but due to their high toxicity and adverse effects, researchers are focusing on natural antitumor drugs with higher efficacy and lower toxicity [46]. A large number of anticancer peptides can be isolated from milk protein, indicating that milk and dairy products are good sources of anticancer peptides [47]. For example, buffalo milk has been shown to exhibit strong inhibitory effects on human HCT116 and Cal 27 cancer cells [48]. For yak milk, research has found that the released peptides from yak milk casein have various biological activities. A study found that the hydrolysates obtained from yak milk casein by trypsin and alkaline protease had strong inhibitory effects on MCF7 and MDA-MB-231 cells. One of the purified nonapeptides, TPVVVPPFL, had the strongest inhibitory effect on these two cancer cells, which could induce apoptosis by blocking the cell cycle of cancer cells [49]. In addition, the lactobacilli isolated from yak milk also showed strong anticancer activity. The Lan4 strain isolated from yak milk could induce apoptosis in Hela cells without toxicity to non-cancerous cells, such as HEK293 [50]. It was also found that the extracellular polysaccharides produced by *Lactobacillus casei* SB27 in yak milk could significantly inhibit the proliferation of HT-29 colon cancer cells. This polysaccharide upregulated the expression of the genes Bad, Bax, Caspase-3, and Caspase-8 in HT-29 cells [51]. These studies demonstrate the potential of yak milk to develop functional foods, and its anticancer peptides and probiotics can serve as natural antitumor drugs. Furthermore, some studies have found that the BgIFITM2 and BgIFITM3 proteins, which are expressed by the yak IFITM2 and IFITM3 genes, have anticancer activity [52,53]. Although BgIFITM3 protein is mainly expressed in the liver and may not be found in yak milk, it does prove that proteins or peptides from yak sources have anticancer activity. The antioxidant and anticancer activities of yak milk and its bioactive components are summarized in Table 8.

### 3.3. Antibacterial Activity

There are three main antibacterial components in yak milk, including yak milk peptides, lactic acid bacteria, and their metabolites. Many studies have confirmed the high antibacterial activity of antibacterial peptides [58]. Research has shown that two highly efficient antibacterial peptides with amino acid sequences of Arg-Val-Met-Phe-Lys-Trp-Ala and Lys-Val-Ile-Ser-Met-Ile were screened from yak milk protein hydrolysate prepared by gastric protease hydrolysis of yak milk protein. These two antibacterial peptides were found to have inhibitory effects on *Bacillus subtilis*, *Staphylcoccus aureus*, *Listeria innocua*, *Escherichia coli*, *Enterobacter cloacae*, and *Salmonella paratyphi*, with the latter also being able to inhibit the growth of fungi [59]. Yak milk casein hydrolysate is also a substance with high antibacterial activity, and research has found that it has a significant inhibitory effect on Escherichia coli and no toxicity on RAW 264.7 cells [60]. Furthermore, the multitude of beneficial microorganisms in yak milk also give it potential antibacterial activity. The Y5-P1 strain isolated from the fermented yak milk showed inhibitory activity against both Gram-positive and Gram-negative bacteria, with a minimum inhibitory concentration ranging from 62.5–250 µg/mL [61]. In addition, in vivo experiments have also shown the antibacterial potential of yak milk lactic acid bacteria. *Lactobacillus reuteri* isolated from yak milk displayed good antibacterial activity in the induced mouse model of *Escherichia coli*. Supplementing the mice with *Lactobacillus reuteri* significantly increased the abundance and diversity of the bacterial community in the duodenum, jejunum, and ileum and alleviated the weight loss caused by *Escherichia coli* [62]. This indicates that probiotics derived from yak milk can improve gut health. Finally, bacteriocins produced by Lactobacillus plantarum SHY 21-2 and exopolysaccharides produced by Streptococcus thermophilus ZJUIDS-2-01 isolated from yak milk yogurt also exhibit good antibacterial properties [63,64]. These antibacterial components of yak milk have potential applications in food and industry.

### 3.4. Antihypertensive Activity

Hypertension is a risk factor for cardiovascular disease and is associated with various diseases such as stroke, heart failure, and myocardial infarction. Biologically active peptides released from food proteins are widely used to treat hypertension, and their action pathways include but are not limited to ACE inhibition [65]. Hypotensive peptides obtained from milk have been found to have benefits in lowering hypertension and improving cardiovascular disease [66]. Similarly, hypotensive peptides with high activity are found in yak milk. The peptide KYIPIQ purified from yak milk casein has strong ACE inhibitory activity and may serve as a source of therapeutic drugs for hypertension [67]. In vitro studies have shown that the peptide KYIPIQ can increase NO synthesis and eNOS expression in human vascular endothelial cells. Hydrolysates of yak milk casein have strong ACE inhibitory activity, with an IC_50_ of 0.38 mg/mL. The amino acid sequences of the purified peptides YQKFPQY, LPQNIPPL, SKVLPVPQK, LPYPYY, and FLPYPYY from yak milk casein hydrolysates match well with known biologically active peptides in cow milk protein [68]. This indicates that yak milk is also a source of antihypertensive peptides. Many studies have characterized the amino acid composition of peptides with ACE inhibitory activity purified from yak milk hydrolyzed proteins. These peptides include PPEIN, PLPLL, SLVYPFPGPI, KFPQY, MPFPKYP, MFPPQ, QWQVL, PFPGPIPN, KYIPIQ, and LPLPLL [24,69,70,71]. As a potential precursor of biologically active peptides, yak milk casein can release ACE inhibitory peptides through single or combined enzyme treatments [72]. The hypotensive activity of yak milk mainly comes from its biologically active peptides, and their antihypertensive effect is manifested through ACE inhibition. The antibacterial and antihypertensive activities of yak milk and its bioactive components are summarized in Table 9.

### 3.5. Antifatigue

The latest in vivo study found that yak collagen peptides exhibit a good anti-fatigue effect in mice [73]. As for yak milk, one study found that orally ingesting yak milk powder can dose-dependently increase the forced swimming time of mice while also increasing their liver glycogen content, decreasing their serum triglyceride levels, and reducing the levels of blood lactate and serum urea nitrogen caused by exercise [74]. This indicates that yak milk can enhance the body endurance of mice and relieve fatigue. *Lactobacillus fermentum* HFY03, isolated from fermented yak milk, has been shown to have anti-fatigue and antioxidant functions in in vivo experiments. After giving ICR mice HFY03 for 4 weeks, their swimming exhaustion time was prolonged, while the content of urea nitrogen and lactate in their bodies decreased and the content of fatty acids and liver glycogen increased, as well as lowering the levels of alanine aminotransferase, creatine kinase, and aspartate aminotransferase in their serum. HFY03 also dose-dependently reduced MDA, CAT, and SOD levels [75]. In short, HFY03’s anti-fatigue characteristics are related to reducing protein breakdown, increasing liver glycogen storage capacity, reducing lactate accumulation, and increasing fat consumption. While there is not much research on the anti-fatigue properties of yak milk, its effectiveness in this regard is indeed significant. In the future, it could be used to develop products with anti-fatigue properties for special groups such as the elderly and athletes.

### 3.6. Improving Constipation

Constipation is a common gastrointestinal disease, and the positive role of probiotics in treating constipation has been extensively studied in both animals and humans [76]. Some studies have shown that probiotics in yak milk have an improving effect on activated carbon-induced constipation in mice. *Lactobacillus fermentum* Lee (LF-Lee), isolated from yak milk, has a positive effect on constipation induced by activated carbon in ICR mice. After oral administration of LF-Lee for nine days, the levels of MTL, Gas, ET, AChE, SP, and VIP in the mouse serum were significantly increased, and SS levels were significantly decreased [77]. Similarly, *Lactobacillus fermentum* YS2 and *Lactobacillus plantarum* YS-3 isolated from yak yogurt can alleviate activated carbon-induced constipation in Kunming mice [78,79].

### 3.7. Cholesterol-Lowering Effects

The cholesterol-lowering effects of probiotics have been widely studied and confirmed [80]. Some probiotics isolated from yak milk and yogurt also have the potential to lower cholesterol. Research shows that *Lactobacillus casei* YBJ02 has an inhibitory effect on the increase in blood lipids in mice with hyperlipidemia. YBJ02 can lower cholesterol in the liver and feces of hyperlipidemic mice, lower serum TG, TC, and LDL-C levels, and increase HDLC levels. The potential mechanism may be to inhibit lipid accumulation by regulating the synthesis of intestinal flora and obesity genes [81]. In addition, *Lactobacillus plantarum* Lp3, found in yak milk, has been found to have higher cholesterol-lowering properties in in vitro experiments. In vivo studies have also found that rats with hyperlipidemia who consume Lp3 can significantly reduce cholesterol and triglyceride levels in both their serum and liver and reduce lipid deposition in liver tissue cytoplasm [82]. These results suggest that YBJ02 and Lp3 may be potential probiotics for the treatment of hyperlipidemia and that yak milk may be a resource for developing cholesterol-lowering drugs.

### 3.8. Anti-Hypoxic

Yak milk is one of the primary sources of nutrition for residents living in high-altitude and hypoxic environments, and research has confirmed its anti-hypoxic activity. Through a hypoxic mouse model, researchers found that yak milk powder can extend the survival time of mice under normal hypoxic conditions and improve their red blood cell and hemoglobin levels. Compared to normal cow’s milk, yak milk has stronger anti-hypoxic activity [83]. In vitro experiments have also found that bta-miR-34a expressed by yak milk extracellular vesicles has a protective effect on IEC-6 survival under hypoxic conditions and may alleviate hypoxic damage to the intestinal tract [84]. These studies suggest that yak milk may be a novel functional food ingredient with anti-hypoxic properties.

### 3.9. Other Functions

It has been discovered that the proteolytic products from yak milk cheese have anti-inflammatory activity. In vitro studies have found that these products can significantly reduce the production of the pro-inflammatory cytokines IL-1β, IL-6, and TNF-α, as well as NO, in peritoneal macrophages stimulated by LPS in mice, demonstrating strong anti-inflammatory effects [55]. The latest research has also found that yak milk extracellular vesicles can reduce the incidence and severity of intestinal inflammation by activating the PI3K/AKT/C3 signaling pathway [85]. In addition to the functions mentioned above, the active components in yak milk also have immunomodulatory activity [25] and have therapeutic effects on osteoporosis and alcoholic liver injury [86,87]. Other functional activities of yak milk and its bioactive components are summarized in Table 10.

## 4. Yak Milk Products

The color of yak milk is milky white, and it has a thick texture and a distinctive yak hair flavor. Therefore, it is generally not consumed directly and is often processed into various yak milk products. On the one hand, this is for longer preservation of yak milk, and on the other hand, it is to obtain products with better flavor and higher nutrition. Pastoralists living on the Qinghai-Tibet Plateau make yak butter from yak milk and use it as a daily food and source of nutrition. Butter is one of the main products of yak milk, and the content of butter in yak milk is about 6%. Yak butter is rich in polyunsaturated fatty acids and therefore has higher health benefits [90]. Pastoralists often make yak butter by natural fermentation and add it to various foods for consumption, such as zanba, pancakes, and fried dishes [16]. Yak cheese is an industrial product made from yak milk. In the 1980s, Nepal first established a yak milk cheese industry. For more than 40 years, yak milk cheese has been produced and used in multiple countries [91]. Today, yak milk cheese products include various types such as *chhurpi*, *chuto*, *hapiruto*, and Cheddar-style yak milk cheese [92]. Most of these cheeses are dry, and only a few have a soft texture. Yak milk cheese generally contains 20–30% water, 30% fat, 25% protein, and about 3.5% ash, and its composition changes with the maturity of the cheese. Yak milk cheese is not only rich in nutrients such as palmitic acid, oleic acid, L-histidine, and active peptides but also has strong antioxidant activity [24,92]. In addition, *Lactobacillus plantarum* separated from yak milk cheese has been found to have strong antibacterial properties, which can cause the death of various pathogenic bacteria by destroying the integrity of the cell membrane [93]. In addition, yak milk is also used to make paneer [94]. Yak milk paneer can be used directly, similar to paneer made from other milks, without additional processing [95]. However, yak milk paneer is not easy to preserve and has a short shelf life. Using vacuum packaging and utilizing predictive models can ensure the food safety of yak milk paneer [96].

Yak yogurt is the most common processed product made from yak milk. It is a natural fermented dairy product made by pastoralists in high-altitude areas with a unique flavor and high nutritional value [97]. Yak milk contains a variety of microorganisms that are beneficial to human health [98]. Studies have found that beneficial bacteria isolated and identified from yak yogurt have multiple functional activities, such as antibacterial [63,99], blood glucose regulation [100], weight loss and lipid-lowering effects [101], and improving gastric ulcers [102]. However, currently, yak yogurt is limited to household production without fixed production standards and has a short shelf life, which makes large-scale production impossible [103,104].

Additionally, casein and bioactive peptides isolated from yak milk are used as raw materials for food additives and functional food products. Moreover, infant formula milk powder supplemented with yak milk is more easily absorbed by the human body and has the characteristics of improving digestive function and low allergenicity [105].

**Figure 3:**
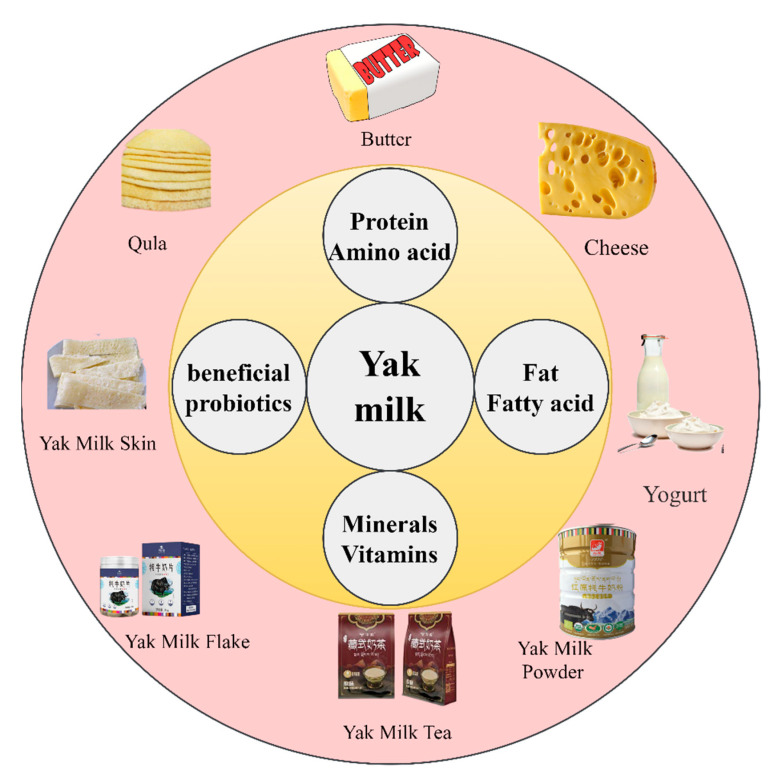
Yak milk products.

In addition to the above-mentioned yak milk products, yak milk is also used to make yak milk powder [106], yak milk skin, yak milk tea, yak milk cake, and so on [16]. Figure 3 shows some yak milk products.

## 5. Conclusions and Prospects

Yak milk is a nutrient-rich food with multiple functional activities. As a unique food in high-altitude regions, yak milk has made a significant contribution to the health of local herdsmen. In this article, we reviewed the nutritional components of yak milk, classified and elaborated on its functional roles and mechanisms, and summarized its related products. It is evident that yak milk contains more nutrients than regular cow milk, has a more reasonable amino acid composition, higher levels of unsaturated fatty acids, and is rich in minerals. The abundant microorganisms in yak milk and its products greatly enhance the functional value of yak milk. Overall, yak milk can be used as a high-nutrition food for the production of dietary supplements and as a raw material for the development of new functional foods and health products.

Although yak milk has gained attention as a new type of milk and significant progress has been made in its nutritional value and functional effects research, some key issues still need to be further explored and resolved. First, it is necessary to strengthen the mechanistic study of the characteristics of yak milk and to explore its special features in depth. Second, it is still necessary to continue exploring the other functional potentials of yak milk and fully realize its health-promoting effect on humans. In addition, more yak milk products, especially functional foods and health products, need to be developed based on the characteristics of yak milk. The prerequisite for these efforts is to ensure the yield and quality of yak milk. 

In general, the development and utilization of yak milk require the widespread application of new technologies and the joint promotion of different disciplines in order to explore in depth the composition and functional role of its components. With the rapid increase in consumer demand for high-quality food, the development of the yak milk industry requires larger scale, deeper processing, and more products. Compared with other mammalian milks, people’s understanding of yak milk is still not comprehensive enough, and its potential characteristics and functional advantages need to be further explored.

## Figures and Tables

**Table 1:** Composition of substances in different types of yak milk (%) [7,16,17].

Yak Breeds (Country)	Milk Solids	Fat	Protein	Lactose	Ash
Tianzhu White yak (China)	16.3–18.4	5.6–5.8	4.7–6.5	5.0–5.3	0.77–0.87
Jiulong (China)	17.3–17.8	6.9–7.2	4.9–4.9	4.7–4.8	0.79–0.83
Maiwa (China)	17.5	6.3	4.9	5.4	0.82
Inner Mongolia (China)	17.8	6.8	5.0	5.1	0.86
Lulang (China)	18.0	6.9	5.0	4.8	0.79
Songduo (China)	18.4	7.1	5.0	5.1	0.81
Milashan (China)	19.0	7.4	5.2	5.2	0.83
Jiali (China)	16.3	6.8	5.0	3.6	0. 95
Pali (China)	16.3	6.0	5.7	3.8	/
Sibu (China)	17.1	7.5	5.3	3.5	/
Kirghizia	17.4	6.6	6.3	4.6	0.87
Nepal	17.4	6.5	5.4	4.6	0.90
India	17.9	6.5	5.9	4.7	0.87
Holsteinbovine	11.8–13.7	2.8–4.0	2.8–4.0	4.6–4.9	0.60–0.80
Yellow bovine (China)	12.8	3.9	3.4	4.8	0.86
Buffalo (China)	18.4	7.6	4.9	4.7	0.85

“/” indicates that the data is not available.

**Table 2:** Nitrogen distribution in different types of yak milk (%) [7,16,22,23].

	Zhongdian Yak	Gannan Yak	Maiwa Yak	Cow	Buffalo	Goat
TN	0.68 ± 0.02	0.84 ± 0.06	0.79 ± 0.04	3.25 ± 0.03	3.87 ± 0.02	2.95 ± 0.02
NPN	0.07 ± 0.11	0.03 ± 0.02	0.04 ± 0.01	0.33 ± 0.03	0.38 ± 0.02	0.39 ± 0.01
NPN/TN	10.29	3.58	5.06	10.15	9.82	13.22
WPN	0.13 ± 0.52	0.17 ± 0.04	0.15 ± 0.01	0.47 ± 0.01	0.68 ± 0.02	0.53 ± 0.02
WPN/TN	19.12	20.23	18.99	14.46	17.57	17.97
CN	0.48 ± 0.17	0.64 ± 0.06	0.60 ± 0.03	2.79 ± 0.02	3.20 ± 0.03	2.44 ± 0.03
CN/TN	70.59	76.19	75.95	85.85	82.69	82.71
WPN/CN	27.08	26.41	25	16.85	21.25	21.72

**Table 3:** Types and contents of proteins in different types of milk [1,10,16].

Types of Proteins	Yak	Cow	Buffalo	Goat
Total casein (g/100 g)	2.10–4.00	2.40–2.80	2.70–5.00	2.30–3.80
αS1-casein (mg/100 g)	416–1024	806–1508	1147–1924	135–1020
αS2-casein (mg/100 g)	288–576	182–390	222–629	270–750
β-casein (mg/100 g)	1184–1632	728–988	1295–1702	1020–1920
κ-casein (mg/100 g)	384–672	234–520	407–592	300–570
Total whey proteins (g/100 g)	1.10	0.50–0.70	0.60–1.00	0.30–1.20
α-lactalbumin (mg/100 g)	77–220	96–150	117–303	85–250
β-lactoglobulin (mg/100 g)	550–946	198–402	301–441	170–385
Serum albumin (mg/100 g)	77–165	36–45	2.1–35	25–110
Lactoferrin (mg/kg)	200–700	20–500	20–300	20–300
Immunoglobulins (mg/kg)	100–400	150–1000	500–1300	150–500
Lactoperoxidase (units/mL)	2.95	1.40	0.90	0.26–4.55

**Table 4:** Amino acid composition and content (g/100 g) of proteins in yak milk [7,16,22,26].

Types of Amino Acids	Maiwa Yak	Gannan Yak	Human	Bovine	Goat
Thr	0.18	0.21	0.05	0.15	0.16
Val	0.25	0.22	0.06	0.16	0.24
Met	0.11	0.13	0.02	0.06	0.08
Ile	0.23	0.20	0.06	0.14	0.21
Leu	0.42	0.46	0.10	0.29	0.31
Phe	0.21	0.23	0.05	0.16	0.16
Lys	0.37	0.39	0.07	0.27	0.29
His	0.11	0.11	0.02	0.10	0.09
Trp	0.06	0.06	0.02	0.05	0.04
EAA	1.94	2.00	0.45	1.33	1.58
Cys	0.03	0.03	0.02	0.02	0.05
Arg	0.15	0.15	0.04	0.11	0.12
Pro	0.45	0.48	0.08	0.32	0.37
Asp	0.33	0.36	0.08	0.26	0.21
Ser	0.23	0.27	0.04	0.16	0.18
Glu	1.03	1.13	0.17	0.77	0.63
Gly	0.09	0.10	0.03	0.06	0.05
Ala	0.14	0.16	0.04	0.10	0.12
Tyr	0.20	0.22	0.05	0.15	0.18
NEAA	2.66	2.92	0.55	1.95	1.91
TAA	4.60	4.91	1.00	3.33	3.49
EAA/NEAA	73%	68%	82%	68%	81%
EAA/TAA	42%	41%	45%	40%	45%

EAA: essential amino acids; NEAA: non-essential amino acids; TAA: total amino acids.

**Table 5:** Fatty acid composition of yak milk (%) [28,29,31,32].

Fatty Acid	Content	Fatty Acid	Content	Fatty Acid	Content
ECSFA		BCSFA		C22:1 c13	0.12 ± 0.06
C4:0	2.12 ± 0.63	C13:0 iso	0.07 ± 0.01	C24:1 c15	0.04 ± 0.02
C6:0	1.49 ± 0.20	C13:0 anteiso	0.02 ± 0.00	∑ MUFA	35.60
C8:0	0.92 ± 0.11	C14:0 iso	0.22 ± 0.03	PUFA	
C10:0	1.75 ± 0.23	C15:0 iso	0.46 ± 0.08	C18:2 c9c12	1.13 ± 0.24
C12:0	1.47 ± 0.26	C15:0 anteiso	0.79 ± 0.13	C18:2 t9t12	0.18 ± 0.02
C14:0	6.25 ± 0.94	C16:0 iso	0.35 ± 0.07	∑ Nonconjugated C18:2 others	0.76 ± 0.14
C16:0	22.06 ± 6.45	C17:0 iso	0.46 ± 0.08	C18:3 c6c9c12	0.02 ± 0.01
C18:0	15.03 ± 2.27	C17:0 anteiso	0.52 ± 0.11	C18:3 c9c12c15	1.12 ± 0.24
C20:0	0.69 ± 0.30	C18:0 iso	0.07 ± 0.03	C18:2 c9t11	2.57 ± 1.17
C22:0	0.35 ± 0.15	∑ BCSFA	2.95	C18:2 t10c12	0.03 ± 0.03
C24:0	0.22 ± 0.07	MUFA		C20:2 c11c14	0.02 ± 0.02
∑ ECSFA	52.33	C10:1 c9	0.31 ± 0.08	C20:3 c8c11c14	0.02 ± 0.02
OCSFA		C12:1 c5	0.00 ± 0.01	C20:4 c5c8c11c14	0.09 ± 0.02
C5:0	0.01 ± 0.01	C12:1 c9	0.03 ± 0.01	C20:3 c11c14c17	0.02 ± 0.01
C7:0	0.02 ± 0.01	C14:1 c9	0.29 ± 0.07	C20:5 c5c8c11c14c17	0.06 ± 0.01
C9:0	0.01 ± 0.01	C16:1 c7	0.27 ± 0.09	C22:2 c13c16	0.02 ± 0.02
C11:0	0.02 ± 0.01	C16:1 c9	1.75 ± 0.44	C22:4 c7c10c13c16	0.03 ± 0.03
C13:0	0.06 ± 0.01	C17:1 c9	0.37 ± 0.13	C22:5 c4c7c10c13c16	0.02 ± 0.02
C15:0	1.31 ± 0.19	∑ C18:1	31.81 ± 7.61	C22:5 c7c10c13c16c19	0.21 ± 0.04
C17:0	0.84 ± 0.18	C19:1 c10	0.26 ± 0.08	C22:6 c4c7c10c13c16c19	0.03 ± 0.01
C19:0	0.17 ± 0.06	C19:1 t10	0.05 ± 0.04	∑ n-6	1.57
C21:0	0.14 ± 0.06	C20:1 c9	0.21 ± 0.08	∑ n-3	1.43
C23:0	0.19 ± 0.06	C20:1 c11	0.07 ± 0.02	n-6/n-3	1.09
∑ OCSFA	2.78	C20:1 t11	0.01 ± 0.01	∑ PUFA	6.33

ECSFA: even-chain saturated fatty acid; OCSFA: odd-chain saturated fatty acid; BCSFA: branched-chain saturated fatty acid; c: cis; t: trans.

**Table 6:** Mineral composition and content (mg/kg) in different types of milk [16,31,33].

	Maiwa Yak	Gannan Yak	Human	Bovine	Goat
Cu	0.45 ± 0.08	0.65 ± 0.02	0.6	0.1–0.6	0.5
Mg	154.10 ± 13.22	150.59 ± 13.98	40	90.00–140.00	160
Zn	7.31 ± 0.44	1.76 ± 0.33	3.8	2.00–6.00	5.6
Fe	0.57 ± 0.04	1.25 ± 0.05	2.0	0.012–0.035	0.7
Mn	0.06 ± 0.01	0.02 ± 0.02	0.7	0.16–0.35	0.32
Ca	1545.45 ± 145.61	1525.2 ± 177.0	330	1000.00–1300.00	1340
P	922.04 ± 70.13	1023.9 ± 81.2	430	900.00–1000.00	1210

**Table 7:** Vitamin composition and content (μg/100 g) in different types of milk [10,16,35].

	Maiwa Yak	Gannan Yak	Human	Bovine	Goat
Vitamin B1	48.54 ± 11.54	23.56 ± 11.29	17.00	45.00	68.00
Vitamin B2	79.49 ± 28.15	20.79 ± 5.74	20.00	175.00	376.00
Vitamin B3	2.61 ± 3.21	1.58 ± 0.67	0.20	90.00	0.41
Vitamin B6	40.75 ± 16.21	0.36 ± 0.18	0.01	0.23	0.08
Vitamin A	13.88 ± 4.52	89.79 ± 4.72	60.80	47.74	46.72
Vitamin D	0.15 ± 0.21	3.95 ± 0.30	0.04	0.06	0.18
Vitamin E	30.15 ± 7.30	91.85 ± 21.25	/	100.00	/

“/” means data is not available.

**Table 8:** The antioxidant and anticancer activities of yak milk and its bioactive components.

Function	Active Ingredients	Cell Line or Model	Dose	Detailed Content	Research Evaluation	References
Antioxidant	Peptide (T10) KALNEINQF	H_2_O_2_-induced injury model of HUVECs	25, 50 or 100 μg/mL	Involved in regulating the Nrf2 signaling pathway and cell apoptosis.	Provide a theoretical basis for the development of functional foods in the future.	[44]
	Peptide (T8) Met-His-Gln-Pro-His-Gln-Pro-Leu-Pro-Pro-Thr-Val-Met-Phe	/	6.25, 12.5, 25 and 50 μg/mL	Improve H_2_O_2_-induced oxidative stress in HUVEC cells by regulating the Nrf2 signaling pathway.	Promote the application of yak milk residue in functional foods.	[54]
	Casein hydrolysate peptide Glu-Leu-Glu-Glu-Leu	/	/	Scavenging activity against superoxide anion and hydroxyl radical (with IC_50_ values of 0.52 and 0.69 mg/mL).	Can serve as a source of natural antioxidant peptides.	[43]
	Casein hydrolysate	/	2.5 mg/mL	Exhibit free radical scavenging activity against DPPH, superoxide, and hydrogen peroxide.	Yak casein hydrolysate exhibits antioxidant activity.	[55]
	*Lactobacillus plantarum* YS4	/	/	Has the ability to scavenge DPPH, ABTS, and hydroxyl free radicals.	The effect was better than that of commercial *Lactobacillus bulgaricus.*	[56]
	Lactic acid bacteria in fermented yak milk	Ageing mice induced by D-galactose	0.3 mL (1 × 10^10^ CFU/mL)	The activity of GSH-Px in livers and serum, as well as the activity of SOD in mouse serum and brains, significantly increased, while the level of MDA in mouse livers and serum significantly decreased.	It is a potential antioxidant strain for the production of functional foods.	[45]
	Casein hydrolysate	/	/	The antioxidant activity of trypsin hydrolysate is the highest.	Yak milk casein can serve as a resource for producing antioxidant peptides.	[57]
	*Lactobacillus plantarum* As21	Caenorhabditis elegans	0.3 mL (1 × 10^8^ CFU/mL)	Reduced the production of ROS and MDA and promoted the production of SOD, CAT, and GSH.	It may be a potential probiotic strain for delaying aging and can be used in functional foods.	[39]
Anti-cancer activity	Lan4 strain	Hela cells	/	Induced maximum apoptosis in Hela cells (87%) and was non-toxic to non-cancerous HEK293 cells.	This shows excellent probiotic properties and ideal health benefits.	[50]
	Peptide TPVVVPPFL	MDA-MB-231 and MCF7 cells	0, 62.5, 125, 250, 500, and 1000 µg/mL	Induces G2-M cell cycle arrest in MCF7 cells, S cell cycle arrest in MDA-MB-231 cells, and induces apoptosis in both cancer cell lines.	Yak milk is a good source of peptides with anti-breast cancer cell activity.	[49]
	Exopolysaccharides produced by *Lactobacillus casei* SB27 from yak milk	HT-29 colorectal cancer cells	/	It can significantly inhibit the proliferation of HT-29 colon cancer cells and upregulate the expression of the Bad, Bax, Caspase-3, and -8 genes.	It has the potential to be a functional food and can be a source of natural anti-tumor drugs.	[51]

“/” means the literature did not mention it.

**Table 9:** The antibacterial and antihypertensive activities of yak milk and its bioactive components.

Function	Active Ingredients	Cell Line or Model	Dose	Detailed Content	Research Evaluation	References
Anti-bacterial activity	Exopolysaccharide (EPS-3A) produced by Streptococcus thermophilus ZJUIDS-2-01	/	8 mg/mL	It has inhibitory effects against *Staphylococcus aureus* CMCC 26003 and *Lactobacillus monocytogenes* CMCC 54007.	It can be developed as a natural alternative to antibiotics.	[63]
	Y5-P1	/	/	Antibacterial compound Y5-P1, from this strain, has biologically active effects against both Gram-positive and Gram-negative bacteria.	It could be further developed as a candidate drug against highly resistant pathogens.	[61]
	Antimicrobial peptides Arg-Val-Met-Phe-Trp-Ala and Val-Ile-Ser-Met-Ile.	/	/	The peptide Arg-Val-Met-Phe-Lys-Trp-Ala has inhibitory effects against *Bacillus subtilis*, *Staphylococcus aureus*, *Listeria innocua*, *Escherichia coli*, *Enterobacter cloacae*, and *Salmonella typhi*. The peptide Lys-Val-Ile-Ser-Met-Ile also has inhibitory effects against fungi.	/	[59]
	κ-casein hydrolysate	/	/	Exhibits strong antibacterial activity against *Escherichia coli.*	It can serve as a potential inhibitor for *Escherichia coli*.	[60]
		Mouse diarrhea model	1 × 10^9^ CFU/day	Regulates intestinal microbiota in an *E. coli*-induced diarrhea mouse model, improving diarrhea.	Pre-supplementation of LR1 can alleviate clinical symptoms caused by E. coli and promote the health of the gut microbiota.	[62]
	*Lactobacillus Plantarum* SHY21-2	/	/	It has a broad spectrum of antibacterial activity against Gram-positive and Gram-negative bacteria as well as fungi.	It has the potential to be used as a biopreservative in food.	[64]
Anti-hypertensive	Peptides in fermented foods with yak milk	/	/	It has strong ACE inhibitory activity.	Natural nutritional products rich in highly active ACE inhibitory peptides can be developed.	[24]
	YQKFPQY LPQNIPPL SKVLPVPQK LPYPYY FLPYPYY	/	/	It has strong ACE inhibitory activity.	Yak cheese protein can be used as a resource for the production of anti-hypertensive peptides.	[68]
	Casein hydrolyzed peptide	/	/	Release of active and non-toxic ACE inhibitory peptides.	Yak cheese protein is a good precursor for the production of many bioactive peptides.	[72]
	Casein hydrolyzed peptide PPEIN, PLPLL	/	/	It has strong ACE inhibitory activity with an IC_50_ of 0.29 ± 0.01 mg/mL and 0.25 ± 0.01 mg/mL, respectively.	It can be used as a raw material for the production of functional foods.	[70]
	Casein hydrolyzed peptide KYIPIQ	/	/	It has strong ACE inhibitory activity with an IC_50_ of 7.28 μM.	May be a valuable source of ACE inhibitory peptides.	[69]
	KYIPIQ	Human vascular endothelial cells	/	Increased synthesis of NO and expression of eNOS in HUVECs.	It has the potential to be used as a therapeutic agent for the treatment of hypertension.	[67]
	Casein hydrolyzed peptide KFPQY	/	/	It has strong ACE inhibitory activity with an IC50 of 12.37 ± 0.43 μM.	/	[71]

“/” means the literature did not mention it.

**Table 10:** Other functional activities of yak milk and its bioactive components.

Function	Active Ingredients	Cell Line or Model	Dose	Detailed Content	Research Evaluation	References
Anti-fatigue	*Lactobacillus fermentum* HFY03	ICR mice	1:0 × 10^8^ and 1:0 × 10^9^ CFU/kg	Reduces protein catabolism, improves hepatic glycogen storage capacity, reduces lactate accumulation, and increases fat consumption.	It can be used as an anti-fatigue, probiotic nutritional supplement.	[75]
	Yak milk powder	Kunming mice	2.6, 5.2, and 7.8 g·kg bw^−1^·day^−1^	Dose-dependent increase in forced swimming time in mice; increased liver glycogen content; and decreased serum triglyceride levels.	It can improve physical endurance and relieve fatigue.	[74]
Improve constipation	*Lactobacillus fermentum* Lee	ICR mice	1 × 10^8^ and 1 × 10^9^ CFU/mL	Increased gastrointestinal transit ratio; increased MTL, Gas, ET, AChE, SP, and VIP levels.	Preventive effect on constipation in mice.	[77]
	*Lactobacillus plantarum* YS2	Kunming mice	1.0 × 10^8^ and 1.0 × 10^9^ cfu/kg of BW	Reduces the time to first black stool defecation and promotes gastrointestinal motility in constipated mice; increases serum MTL, gastrin, AChE, SP, and VIP levels.	Ability to reduce activated charcoal-induced constipation in Kunming mice.	[78]
	*Lactobacillus plantarum* YS3	Kunming mice	1 × 10^8^ and 1 × 10^9^ CFU/kg	Reduced time required for first black stool evacuation; increased MTL, Gas, ET, AChE, SP, and VIP levels.	It can effectively suppress constipation.	[79]
Hypolipidemic effect	*Lactobacillus plantarum* Lp3	Sprague-Dawley rats	10^9^ CFU/mL	It significantly reduced serum and liver cholesterol and triglyceride levels and reduced lipid deposition in the cytoplasm of rat liver tissue.	May be a potential probiotic for the treatment of hyperlipidemia and may be used in functional foods.	[82]
	*Lactobacillus Casei* YBJ02	Kunming mice	1 × 10^10^ and 1 × 10^9^ CFU/kg	Regulates intestinal flora in mice and inhibits lipid accumulation by regulating obesity gene synthesis through the PPAR-alpha pathway.	It can be used as a probiotic due to its antilipidemic effect.	[81]
Hypoxia resistant	Yak milk powder	BALB/c mice hypoxia model	2.6, 5.2, 7.8 mg·g^−1^ bw·day^−1^	Prolongs survival time under conditions of normoxia and sodium nitrite intoxication; increases red blood cell and hemoglobin levels.	It can be used as a novel anti-hypoxia functional food ingredient.	[83]
	Yak milk exosome miRNA, bta-miR-34a	Intestinal epithelial cells	/	Protective effect on the survival of intestinal epithelial cells under hypoxic conditions.	Further study of the effects of lactogenic exosomal miRNAs on human intestinal health provides a scientific basis.	[84]
Anti-inflammatory	Exosomal proteins	Intestinal epithelial cells 6	0, 50, 100 and 200 ng/μL	Activating the PI3K/AKT/C3 signaling pathway, thereby reducing the incidence and severity of intestinal inflammation.	May be a potential innovative treatment option for intestinal inflammation.	[85]
	Casein hydrolysate	Mouse macrophages	/	Significantly reduced the production of NO and pro-inflammatory cytokines IL-1β, IL-6, and TNF-α in mouse macrophages.	May be used to prevent inflammation-related diseases.	[55]
Improve osteoporosis	Yak Milk	C57BL/6J mice	1000 and 2000 mg/kg/d	Can improve bone mass and microarchitecture by inhibiting bone resorption in osteoporotic mice.	/	[86]
Improve liver injury	*Lactobacillus plantarum* HFY05	Mouse model of alcoholic liver injury	/	Reduced AST, ALT, ALP, TG, TC, BUN, NO, and MDA levels and up-regulated ALB, SOD, CAT, and GSH-Px levels in the serum of liver-injured mice; down-regulated IL-6, IL-12, TNF-α, and IFN-γ levels in the serum of liver-injured mice; and regulated intestinal flora.	Hepatoprotective effect on alcoholic liver injury in experimental mice.	[87]
Immune regulation	Casein hydrolysate	Mouse spleen cells	/	Promotes lymphoproliferative activity and IL-2 production in mouse splenocytes.	Casein protease hydrolysate has high immunomodulatory activity.	[25]
Neuroprotective effect	Fermented yak milk peptide LYLKPR	H_2_O_2_ damage in HT-22 cells	25, 50, 100, 150, and 200 μM	LYLKPR ameliorates oxidative stress-mediated neuronal injury by inhibiting the NLRP3 inflammasome through regulation of the Nrf2/Keap-1/HO-1 pathway.	Helps with anti-aging.	[88]
Cholesterol regulation	*Lactobacillus plantarum* LP3	Sprague-Dawley rats	1 × 10^10^ CFU/mL	Reduces high cholesterol induced by a high-fat diet by modulating intestinal microbiota and metabolites.	*Lactobacillus plantarum* LP3 has good potential as a therapeutic probiotic.	[89]

“/” means the literature did not mention it.

## Data Availability

Not applicable.
